# Citation and adherence to TRIPOD guidelines by published radiological prognostic models: systematic review

**DOI:** 10.1093/bjr/tqaf174

**Published:** 2025-07-24

**Authors:** Maira Hameed, Louis Dwyer-Hemmings, Jason K C Mak, William Weston, Stuart A Taylor, Sue Mallett, Steve Halligan

**Affiliations:** Centre for Medical Imaging, University College London UCL, London, WC1E 6JF, United Kingdom; Department of Radiology, University College Hospital, London, NW1 2BU, United Kingdom; Centre for Medical Imaging, University College London UCL, London, WC1E 6JF, United Kingdom; Department of Radiology, University College Hospital, London, NW1 2BU, United Kingdom; Department of Radiology, University College Hospital, London, NW1 2BU, United Kingdom; Department of Radiology, University College Hospital, London, NW1 2BU, United Kingdom; Centre for Medical Imaging, University College London UCL, London, WC1E 6JF, United Kingdom; Department of Radiology, University College Hospital, London, NW1 2BU, United Kingdom; Centre for Medical Imaging, University College London UCL, London, WC1E 6JF, United Kingdom; Centre for Medical Imaging, University College London UCL, London, WC1E 6JF, United Kingdom; Department of Radiology, University College Hospital, London, NW1 2BU, United Kingdom

**Keywords:** prognostic, models, statistical, regression analysis, multivariate analysis, radiology

## Abstract

**Objectives:**

We aimed to establish the extent to which prognostic models published in highly indexed radiological journals cite and adhere to generally accepted reporting guidelines.

**Methods:**

We identified articles reporting multivariable prognostic models, developed using regression and published in the top 3 indexed general radiological journals, December 2022 to May 2023 inclusive. We determined whether they cited the generally accepted reporting guideline, Transparent Reporting of a multivariable prediction model for Individual Prognosis or Diagnosis (TRIPOD). We scored adherence to individual TRIPOD domains to determine reporting quality both overall and for specific areas.

**Results:**

We included 140 articles. Only 4% (*n* = 6) cited TRIPOD, with just 1 including the checklist. TRIPOD adherence was poor overall, with a median score of 57% (interquartile range, IQR 48% to 64%, range 30% to 87%). Individual domains particularly poorly reported were, title (2% adherence), abstract (3%), and statistical analysis (5%). Only 38% articles (*n* = 53) named a statistician author. Only one journal mentioned TRIPOD guidelines in their “Instructions For Authors” but did not mandate checklist submission.

**Conclusions:**

The large majority of prognostic models published in highly indexed radiological journals did not cite TRIPOD, nor fulfil its recommendations.

**Advances in knowledge:**

Authors should adhere to the TRIPOD statement so that their work is reported with sufficient clarity, and radiological journals should stipulate adherence for authors submitting prognostic models for publication.

## Background

Multivariable prognostic models aim to evaluate the probability of a future event and now comprise a large proportion of indexed radiological literature.^1^ Model publication has increased greatly, facilitated by easy data access and inexpensive computational power.^2^ However, despite increasing publication, the methodological quality of prognostic models appears poor.^2^ A recent systematic review found that most models reported in major radiological journals did not include an internal evaluation and omitted data that would enable other researchers to evaluate the model.^1^ Clinicians will not use models that are poorly developed and reported inadequately.^3^

The Transparent Reporting of a multivariable prediction model for Individual Prognosis or Diagnosis (TRIPOD) statement, published 2015, quantifies reporting quality of prognostic models.^4^^,^^5^ TRIPOD uses a checklist, similar to Consolidated Standards of Reporting Trials (CONSORT) for randomized controlled trials^6^ and Preferred Reporting terms for Systematic Reviews and Meta-analyses (PRISMA) for systematic reviews,^7^ and is included in the Enhancing the Quality and Transparency of health Research (EQUATOR) database of reporting guidelines.^8^ The TRIPOD checklist stipulates items for reporting, ensuring the research is transparent and that other workers can assess methodological quality, risk of bias, generalizability, and even evaluate the model themselves. Since TRIPOD is well-established, we hypothesized it would indicate methodological quality. Via systematic review, we aimed to determine the proportion of prognostic models, published in major radiological journals, citing TRIPOD. We also hypothesized that research citing TRIPOD would be methodologically superior to research that did not, and therefore intended to compare adherence between these two groups. Finally, we assessed if major radiological journals mandated TRIPOD checklist submission.

## Methods

Ethical approval is not required for secondary research using primary literature. Our review is reported according to PRISMA.^9^ The review was not registered, as solely methodological reviews are ineligible.

### Inclusion/exclusion criteria

We searched for articles reporting multivariable prognostic models developed using traditional regression. We excluded models using machine learning/artificial intelligence (AI) since our scoping review suggested these were uncommon. However, non-regression methods to identify candidate predictors were eligible if the model equation was developed using regression. Eligible articles could describe model development, and/or external evaluation (“validation”) and/or updating existing models.

### Information sources

We developed our protocol with a medical statistician (SM) who considered a sample size of approximately 100 articles would provide representative data regarding our primary outcome (ie, the proportion of eligible literature citing TRIPOD). A scoping review found a large volume of eligible research, so we limited our time-horizon to 6 months, excluded diagnostic models, and restricted to the top 3 indexed (Scopus 2022) general clinical radiology journals publishing original research: Radiology, Investigative Radiology, and European Radiology. We anticipated greater methodological quality here than lesser-indexed journals, meaning our findings would likely be a “best-estimate.” All radiological subspecialties were eligible.

### Search strategy, data screening, and extraction

The first author accessed journals’ “table of contents” for individual monthly “print” issues online, from December 2022 to May 2023 inclusive. She identified model reports from titles and abstracts, with eligibility confirmed via the full text. Uncertainty was resolved by discussion with the senior author (SH). Because TRIPOD stipulates 37 individual items, data extraction was shared across 4 radiologists (MH, LD-H, JKCM, WW), with pairs extracting each individual article independently. They extracted the clinical area and outcome assessed, imaging modality, model type (linear/logistic/Cox), and whether machine learning selected predictors. They noted whether TRIPOD was cited, including within Supplementary Material where present. They searched for the TRIPOD checklist. “Instructions for Authors” were reviewed to establish if TRIPOD was stipulated at submission, recommended, or not mentioned.

Researchers then applied the TRIPOD checklist and adherence extraction form (see Supplementary Information 1 and 2), divided into title, abstract, methodology, results, and discussion sections, providing a maximum of 37 individual domains. Adherence for each domain was then scored 0 or 1, recording the reason for 0 scores. A uniform approach was adopted, using published guidance.^5^ Discrepancy was resolved by face-to-face discussion, including senior author review if necessary.

Author lists, published affiliations, author contributions, and acknowledgements were reviewed to establish formal statistical involvement. We also considered whether the model answered a clinically useful question, with the final decision falling to 2 senior radiologists (SAT, SH). Examples included where the model outcome would be obtained elsewhere more reliably in usual practice, or where prediction did not impact clinical decision-making.

### Risk of bias

Because our review assessed guideline adherence and not methodological quality for meta-analysis, no risk of bias assessment was performed.

### Analysis

Extracted data for individual items were expressed as descriptive summary statistics including median, interquartile range (IQR), and range. A percentage adherence score was obtained by dividing the sum of individual TRIPOD items adhered to by each article, divided by the total number of applicable items for that article. Not all 37 TRIPOD domains and elements were relevant to each study; for example, 6 domains apply only to external evaluations. Additionally, 5 items (5c, 10e, 11, 14b, 17) may not apply to specific models.^10^ We determined the numerical difference in adherence scores between studies that did and did not cite TRIPOD. Because overall scores do not identify areas of specific deficiency, we presented results split by reporting domain and graphically. We calculated difference in adherence score for studies including and not including a named statistician. Any difference in TRIPOD citation and adherence scores between journals that suggest/stipulate TRIPOD adherence was determined.

## Results

**Figure 1.**
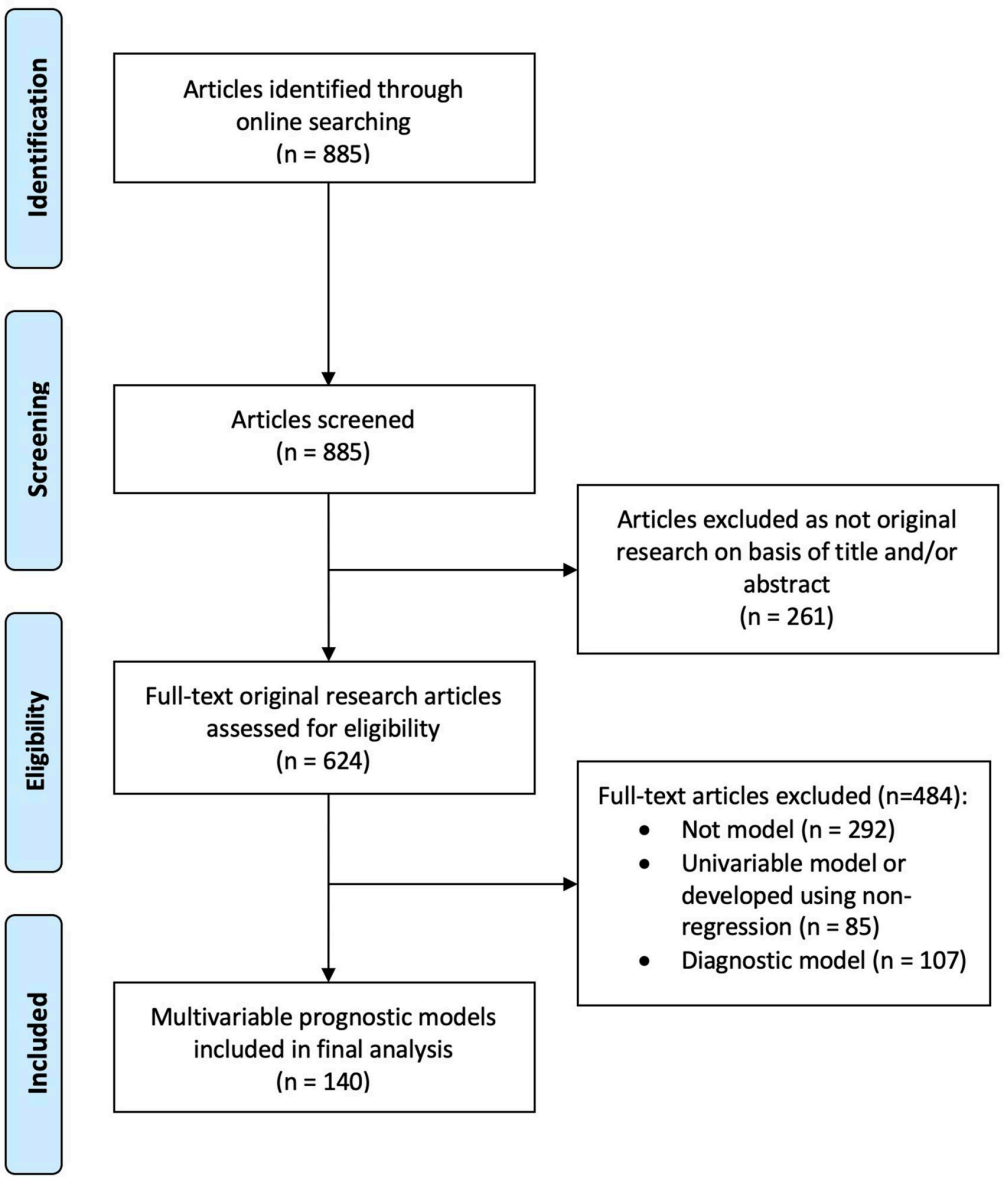
PRISMA diagram of article selection for the systematic review. Abbreviation: PRISMA = Preferred reporting terms for systematic reviews and meta-analyses.

The PRISMA flowchart is presented in Figure 1. The 3 journals published 885 articles during the search period, of which 71% (*n* = 624) described original research (Table 1). Of these, 53% (*n* = 332) described models developed using any technique. Of the 332 articles describing models, 26% (*n* = 85) were univariable (*n* = 7) or developed using nonregression (*n* = 78), and 32% (*n* = 107) were diagnostic, leaving 140 42% (*n* = 140) articles describing multivariable prognostic models (Figure 1, see Supplementary Information 3 for all included studies). All 140 studies developed new models. None externally evaluated or updated previously published models.

**Table 1.** Table showing all articles screened with the respective number of original research articles; the proportion of articles describing models of any description; the proportion of articles describing multivariable models developed using regression; and the proportion of articles describing multivariable prognostic models developed using regression.

Journal	All published articles (no.)	Original research articles (no.)	Original research articles describing models (no. %)	Multivariable models developed via regression (no. %)	Multivariable prognostic models developed via regression (no. %)
Radiology	387	164	90 (55%)	69 (42%)	42 (26%)
Investigative Radiology	47	39	7 (22%	4 (13%)	2 (6%)
European Radiology	451	421	235 (56%)	174 (41%)	96 (23%)
Total	885	624	332 (55%)	247 (41%)	140 (22%)

Study characteristics are described in Supplementary Information 4. The clinical area most frequently assessed was hepatopancreaticobiliary with 24% (*n* = 34) published studies, of which 19 related to hepatocellular carcinoma prognosis. Remaining clinical areas were; 16% (*n* = 22) thoracic (9 lung cancer), 14% (*n* = 19) cardiac, 14% (*n* = 19) neurological (8 stroke), 7% (*n* = 10) breast, 6% (*n* = 9) musculoskeletal, 6% (*n* = 8) gastrointestinal, 6% (*n* = 8) head and neck, 4% (*n* = 5) genitourinary, 3% (*n* = 4) miscellaneous, 1% (*n* = 2) paediatric. CT was the modality most investigated (41%, *n* = 58 studies), followed by MRI (39%, *n* = 55), image guided intervention (8%, *n* = 11), PET (7%, *n* = 10), ultrasound (4%, *n* = 6), radiographs (4%, *n* = 6). 6 studies included multiple imaging modalities.

50% (*n* = 70) articles described Cox models, 42% (*n* = 59) logistic, 7% (*n* = 10) linear, and one omitted the approach used.^11^ Three studies used multiple regression types. 19% (*n* = 26) studies used radiomics, deep learning and/or machine learning to identify candidate predictors but then developed the model using traditional regression. Most of these studies (88%, *n* = 23) used titles such as, “radiomic model to predict,” “deep learning model,” but did not state the final model was developed using traditional regression.

### Journal requirements regarding the TRIPOD statement

Of the three journals searched, instructions for authors for European Radiology stated, “According to the type of study, you are advised to consult the following abstract checklists…TRIPOD.” The checklist was not mandated, however. The other journals did not mention TRIPOD at the time of our review.

### TRIPOD statement citation

Just 6 (4%) of the 140 studies cited TRIPOD^12–17^; 4/96 (4%) in European Radiology (despite TRIPODs mention in their submission guidelines), and 2/42 (5%) in Radiology. In five of these studies, TRIPOD was cited in the methodology section, and in both the methods and results section in the remaining manuscript. Only one study included the TRIPOD checklist (as a Supplementary Table).^17^ Three studies citing TRIPOD named a statistician author.^12–14^

### TRIPOD guideline adherence

**Figure 2.**
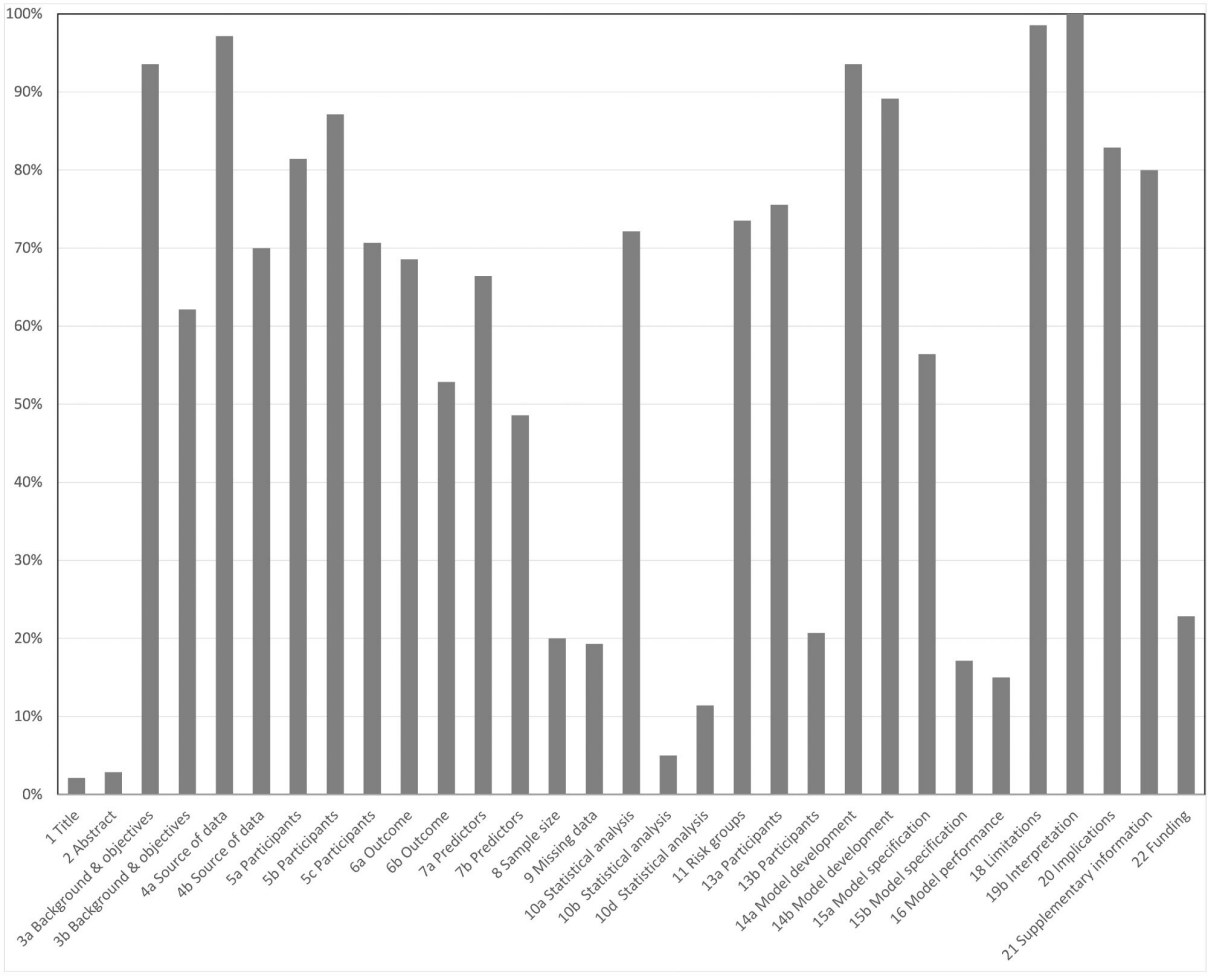
Bar chart showing percentage adherence of selected studies to main TRIPOD domains. As per TRIPOD stipulations, adherence was only positive when all elements within an individual domain were reported. For all domains, the denominator is 140, excepting individual studies to which all domains may not apply. Domains specific to model evaluation/validation were not included as no study reported a genuine external evaluation. Abbreviation: TRIPOD = transparent reporting of a multivariable prediction model for individual prognosis or diagnosis.

The median overall TRIPOD adherence score was 57% (IQR 48% to 64%), range 30%^18^ to 87%.^14^ For the 6 studies citing TRIPOD, median adherence score was 64% (IQR 60% to 74%), range 52% to 87% (the low sample size precluded statistical hypothesis testing). The adherence score was 52% for the single study publishing the checklist.^17^  Figure 2 illustrates adherence for individual domains (a table is presented in Supplementary Information 5). Domain adherence varied widely, with only 5 domains scoring 90% or above: Background/objectives; data sources; model development; limitations; interpretation. Three domains scored less than 10%: Title; Abstract; statistical analysis. Statistical detail was generally reported poorly, especially details of model building procedures (including predictor selection) and sample size calculation (fulfilled by just 20% of articles). 20% (*n* = 28) articles described internal evaluation. Few studies described plans to assess the developed model, notably discrimination and calibration measures. Only 19% (*n* = 27) studies reported how missing data were handled. A worked example, online calculator, or scoring rule was provided by just 17% (*n* = 24) studies. While most studies acknowledged funders, only a minority reported their specific role.

### Statistician authorship

Fifty-three of 140 (38%) studies named a statistician (51 as co-author and 2 as acknowledgement). Their mean overall TRIPOD adherence score was 58% (IQR 48%-65%, range 39%-87%) versus 55% (IQR 49%-61%, range 30%-81%) for studies not naming a statistician.

### Does the model answer a clinically useful question?

Sixteen of 140 (11%) studies were judged to not answer a clinically useful question. For example, a model was developed to predict atrial fibrillation from CT,^19^ a clinical sign that is more simply and reliably determined by taking the patient’s pulse.

## Discussion

Multivariable models that predict a future patient event now comprise a large proportion of indexed radiological research.^1^ Model development and subsequent evaluation requires considerable statistical expertise to avoid bias, which is manifest as “overfitting.”^3^^,^^20^^,^^21^ Poorly developed models will not work in representative patients and will therefore either go unused or possibly harm patients if inaccurate predictions are acted upon. The TRIPOD statement improves research clarity by obliging authors to report information across domains describing model development, evaluation, and implementation: 22 individual aspects that authors should describe in their manuscript are stipulated, some of which are subdivided further, so that the checklist lists 37 individual “items".^4^ The accompanying elaboration document explains why these are important.^5^ For example, the Methods domain (4a) asks authors to describe data sources because spectrum bias influences model generalizability; the Results domain (15b) asks authors explain how to use the model. Not all items will be applicable to all models. For example, if the submitted model does not present an external evaluation, then items related to this (10c, 10e, 12, 13c, 17, 19a) are omitted. Similar comments apply to models that are not updated (items 10e and 17); our search identified no model that was updated.

Reporting guidelines can benefit researchers by alerting them to important aspects of study design, and it is advisable to consult guidelines at the research design stage. It is also known that guideline citation is a marker of research quality.^8^^,^^22^ We had noticed that reports of radiological models usually omitted TRIPOD citation, and decided to investigate this formally via systematic review. Even then, we were very surprised that only 6 (4%) eligible studies cited TRIPOD. This means that most radiologists publishing models are either unaware of TRIPOD or choose simply not to cite it. Some inexperienced authors may first encounter reporting guidelines if their submission is stipulated by a journal. For example, the highest indexed general medical journals mandate guideline checklist submission, and return work unreviewed if authors do not do this. Of the journals we reviewed, only European Radiology mentioned TRIPOD in their author guidelines. However, checklist submission was not mandated and the large majority of reports published during our search period chose to ignore journal advice. Because the British Journal of Radiology mandates TRIPOD checklist submission, we would expect all relevant articles published there to cite TRIPOD and include the checklist. Assessing author guideline awareness for such journals will not be representative because authors are obliged to comply. We believe this is the right approach and that radiological journals should mandate checklist submission. This is especially important since prognostic models now comprise a large proportion of published research; we found that over 40% of original research articles published in Radiology and European Radiology described multivariable models. TRIPOD was published originally in 11 indexed journals. While none were radiological, with over 9000 citations at the time of writing, we would expect imaging researchers to be aware of it. Given this, poor guideline adherence is certainly not restricted to radiological journals.^23^^,^^24^

Ultimately, reporting transparency is more important than guideline citation, so we also investigated adherence. A mean overall adherence score of 57% meant that, on average, around 40% of domains were reported inadequately. This figure is almost identical to the 57.8% found by Park et al^25^ who investigated TRIPOD adherence in 77 radiomics articles. We included any multivariable prognostic model using imaging and also investigated citation in addition to adherence. Because overall adherence does not identify specific deficiencies, we reported adherence for individual domains. Although we initially intended to compare adherence for articles citing and not citing TRIPOD, the tiny number of citations prevented sensible statistical comparison. Authors best reported their objectives, data sources, development, interpretation and limitations. The “Title” domain scored less than 10%, with most articles omitting elements of “prediction” or “risk,” rendering them difficult to identify as prognostic models from title alone. Authors should describe study design accurately.^26^ Similarly, most abstracts omitted details stipulated by TRIPOD, for example, predictors, sample size, and statistical analysis. Articles generally reported statistical details poorly, notably how predictors were selected and measured, and whether missing data were present and, if so, how they were handled (eg, by whole-case analysis or imputation). Online publication now allows considerable Supplementary Material, so word-count restriction is no longer a barrier to full descriptions of statistical procedures. Statisticians consider model development complex, but over 60% of studies had no statistician co-author or acknowledgement. European Radiology requires “Statistics and Biometry” disclosures from authors but, surprisingly, we found some authors declared, “No complex statistical methods were necessary.” Examples included models to predict atrial fibrillation^19^ and congenital diaphragmatic hernia,^27^ both of which combed multiple clinical and imaging factors, clearly indicating complex statistical methods. While some authors may be unaware of the complexity of model development and need for statistical assistance, others may be unable to access statistical expertise; A recent UK survey found that most radiology trainees could not access statistical support.^28^ In these circumstances, it is preferable to not perform the research rather than run the risk of publishing potentially inaccurate and misleading results.^29^

Only 20% of eligible articles described model evaluation and few mentioned plans to do this, for example, by discrimination and calibration. Clinicians will only use models proven effective in representative patients and easy to use in clinical practice.^21^^,^^30^^,^^31^ Few studies presented practical worked examples (TRIPOD domain 15b): a recent systematic review of radiological models found that the model equation was usually omitted (domain 15a).^1^ Most studies presented univariable regression coefficients only, but it is well-established that the causality of these variables cannot be determined from this information alone, because details of mutual adjustment for other variables is absent, a phenomenon known as the “Table 2 fallacy.”^32^

We found no research that externally evaluated or updated a previously published model. Instead, all articles described de-novo development of a new model. We were disappointed by this finding because statisticians generally believe it is more efficient to build on existing models rather than continually develop new models.^21^ A recent systematic review reported that most published radiological models are never externally evaluated.^1^ TRIPOD domain 22 stipulates that authors declare funding roles, for readers to assess external influence, but only a minority reported their specific role; of 42 studies published in Radiology declaring external funding, none described their role.

Our study has limitations. Searching and extracting all indexed radiological journals would have been prohibitive, so we restricted both time-horizon and journals, hypothesizing that the highest-indexed journals would publish best practice, and citation rates climb with time (TRIPOD was published in 2015). Our data therefore likely represent “best practice” for radiology subspecialty journals, which is disappointing given the limited citation and adherence we encountered. It is possible that author guidance at the time of original submission differed from the time of publication. Our online search was restricted to “print” issues, so “online-first” articles will have been excluded. Because diagnosis and prognosis differ,^33^ we excluded diagnostic models: Prognostic models are more challenging since development requires future outcomes rather than existing reference data. We excluded models developed using AI since model predictors and equations are frequently obscure.^34^ While multiple guidelines can apply to a single study, we focused on multivariable prognostic models developed using traditional regression. For example, both TRIPOD and Checklist for Evaluation of Radiomics Research,^35^ may apply to a radiomics study. TRIPOD+AI was published subsequent to our search.^34^

In summary, most descriptions of prognostic models published in highly indexed radiological journals did not cite TRIPOD, the relevant reporting guideline, nor fulfil its recommendations. Authors should adhere to TRIPOD so as to report their work with clarity. Radiological journals should mandate adherence for authors submitting prognostic models.

## Supplementary Material

tqaf174_Supplementary_Data
