# Supplementary material for: Citation and adherence to TRIPOD guidelines by published radiological prognostic models: systematic review
Source: Br J Radiol. 2025 Jul 24;98(1175):1931–7. doi: 10.1093/bjr/tqaf174 (PMC12659733; doi:10.1093/bjr/tqaf174)

**
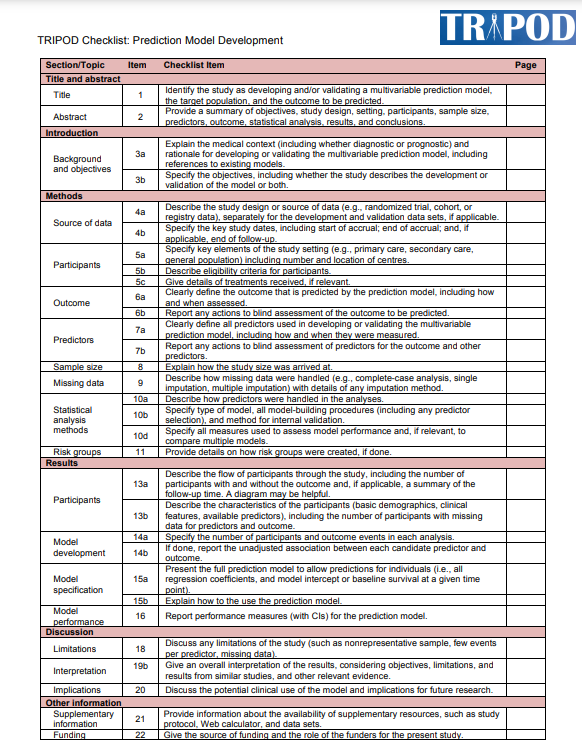
Supplementary information 1:** Transparent Reporting of a multivariable prediction model for Individual Prognosis or Diagnosis (TRIPOD) checklist.

**Supplementary information 2:** TRIPOD Adherence Assessment Form for model development.

|  | **Y=yes; N=no; R=referenced; NA=not applicable** | **Development [D]** |
| --- | --- | --- |
| **Title and abstract** | |  |
| **1** | **Identify the study as developing and/or validating a multivariable prediction model, the target population, and the outcome to be predicted.** |  |
| i | The words developing/development, validation/validating, incremental/added value (or synonyms) are reported in the title |  |
| ii | The words prediction, risk prediction, prediction model, risk models, prognostic models, prognostic indices, risk scores (or synonyms) are reported in the title |  |
| iii | The target population is reported in the title |  |
| iv | The outcome to be predicted is reported in the title |  |
| **2** | **Provide a summary of objectives, study design, setting, participants, sample size, predictors, outcome, statistical analysis, results, and conclusions.** |  |
| i | The objectives are reported in the abstract |  |
| ii | Sources of data are reported in the abstract *E.g. Prospective cohort, registry data, RCT data.* |  |
| iii | The setting is reported in the abstract *E.g. Primary care, secondary care, general population, adult care, or paediatric care. The setting should be reported for both the development and validation datasets, if applicable.* |  |
| iv | A general definition of the study participants is reported in the abstract *E.g. patients with suspicion of certain disease, patients with a specific disease, or general eligibility criteria.* |  |
| v | The overall sample size is reported in the abstract |  |
| vi | The number of events (or % outcome together with overall sample size) is reported in the abstract *If a continuous outcome was studied, score Not applicable (NA)*. |  |
| vii | Predictors included in the final model are reported in the abstract. For validation studies of well-known models, at least the name/acronym of the validated model is reported *Broad descriptions are sufficient, e.g. ‘all information from patient history and physical examination’. Check in the main text whether all predictors of the final model are indeed reported in the abstract.* |  |
| viii | The outcome is reported in the abstract |  |
| ix | Statistical methods are described in the abstract *For model development, at least the type of statistical model should be reported. For validation studies a quote like “model’s discrimination and calibration was assessed” is considered adequate. If done, methods of updating should be reported.* |  |
| x | Results for model discrimination are reported in the abstract *This should be reported separately for development and validation if a study includes both development and validation.* |  |
| xi | Results for model calibration are reported in the abstract *This should be reported separately for development and validation if a study includes both development and validation.* |  |
| xii | Conclusions are reported in the abstract *In publications addressing both model development and validation, there is no need for separate conclusions for both; one conclusion is sufficient.* |  |
| **3a** | **Explain the medical context (including whether diagnostic or prognostic) and rationale for developing or validating the multivariable prediction model, including references to existing models.** |  |
| i | The background and rationale are presented |  |
| ii | Reference to existing models is included (or stated that there are no existing models) |  |
| **3b** | **Specify the objectives, including whether the study describes the development or validation of the model or both.** |  |
| i | It is stated whether the study describes development and/or validation and/or incremental (added) value |  |
| **Methods** | |  |
| **4a** | **Describe the study design or source of data (e.g., randomized trial, cohort, or registry data), separately for the development and validation data sets, if applicable.** |  |
| i | The study design/source of data is described *E.g. Prospectively designed, existing cohort, existing RCT, registry/medical records, case control, case series. This needs to be explicitly reported; reference to this information in another article alone is insufficient.* |  |
| **4b** | **Specify the key study dates, including start of accrual; end of accrual; and, if applicable, end of follow-up.** |  |
| i | The starting date of accrual is reported |  |
| ii | The end date of accrual is reported |  |
| iii | The length of follow-up and prediction horizon/time frame are reported, if applicable *E.g. “Patients were followed from baseline for 10 years“ and “10-year prediction of…”; notably for prognostic studies with long term follow-up. If this is not applicable for an article (i.e. diagnostic study or no follow-up), then score Not applicable (NA).* |  |
| **5a** | **Specify key elements of the study setting (e.g., primary care, secondary care, general population) including number and location of centres.** |  |
| i | The study setting is reported (e.g. primary care, secondary care, general population) *E.g.: ‘surgery for endometrial cancer patients’ is considered to be enough information about the study setting.* |  |
| ii | The number of centres involved is reported *If the number is not reported explicitly, but can be concluded from the name of the centre/centres, or if clearly a single centre study, score Yes.* |  |
| iii | The geographical location (at least country) of centres involved is reported *If no geographical location is specified, but the location can be concluded from the name of the centre(s), score Yes.* |  |
| **5b** | **Describe eligibility criteria for participants.** |  |
| i | In-/exclusion criteria are stated *These should explicitly be stated. Reasons for exclusion only described in a patient flow is not sufficient.* |  |
| **5c** | **Give details of treatments received, if relevant.** *(i.e. notably for prognostic studies with long term follow-up)* |  |
| i | Details of any treatments received are described  *This item is notably for prognostic modelling studies and is about treatment at baseline or during follow-up. The ‘if relevant’ judgment of treatment requires clinical knowledge and interpretation.  If you are certain that treatment was not relevant, e.g. in some diagnostic model studies, score Not applicable.* |  |
| **6a** | **Clearly define the outcome that is predicted by the prediction model, including how and when assessed.** |  |
| i | The outcome definition is clearly presented *This should be reported separately for development and validation if a publication includes both.* |  |
| ii | It is described how outcome was assessed (including all elements of any composite, for example CVD [e.g. MI, HF, stroke]). |  |
| iii | It is described when the outcome was assessed (time point(s) since T0) |  |
| **6b** | **Report any actions to blind assessment of the outcome to be predicted.** |  |
| i | Actions to blind assessment of outcome to be predicted are reported *If it is clearly a non-issue (e.g. all-cause mortality or an outcome not requiring interpretation), score Yes. In all other instances, an explicit mention is expected*. |  |
| **7a** | **Clearly define all predictors used in developing or validating the multivariable prediction model, including how and when they were measured.** |  |
| i | All predictors are reported *For development, “all predictors” refers to all predictors that potentially could have been included in the ‘final’ model (including those considered in any univariable analyses). For validation, “all predictors” means the predictors in the model being evaluated.* |  |
| ii | Predictor definitions are clearly presented |  |
| iii | It is clearly described how the predictors were measured |  |
| iv | It is clearly described when the predictors were measured |  |
| **7b** | **Report any actions to blind assessment of predictors for the outcome and other predictors.** |  |
| i | It is clearly described whether predictor assessments were blinded for outcome *For predictors for which it is clearly a non-issue (e.g. automatic blood pressure measurement, age, sex) and for instances where the predictors were clearly assessed before outcome assessment, score Yes. For all other predictors an explicit mention is expected.* |  |
| ii | It is clearly described whether predictor assessments were blinded for the other predictors |  |
| **8** | **Explain how the study size was arrived at.** |  |
| i | It is explained how the study size was arrived at *Is there any mention of sample size, e.g. whether this was done on statistical grounds or practical/logistical grounds (e.g. an existing study cohort or data set of a RCT was used)?* |  |
| **9** | **Describe how missing data were handled (e.g., complete-case analysis, single imputation, multiple imputation) with details of any imputation method.** |  |
| i | The method for handling missing data (predictors and outcome) is mentioned *E.g. Complete case (explicit mention that individuals with missing values have been excluded), single imputation, multiple imputation, mean/median imputation. If there is no missing data, there should be an explicit mention that there is no missing data for all predictors and outcome. If so, score Yes. If it is unclear whether there is missing data (from e.g. the reported methods or results), score No. If it is clear there is missing data, but the method for handling missing data is unclear, score No.* |  |
| ii | If missing data were imputed, details of the software used are given *When under 9i explicit mentioning of no missing data, complete case analysis or no imputation applied, score Not applicable.* |  |
| iii | If missing data were imputed, a description of which variables were included in the imputation procedure is given *When under 9i explicit mentioning of no missing data, complete case analysis or no imputation applied, score Not applicable.* |  |
| iv | If multiple imputation was used, the number of imputations is reported *When under 9i explicit mentioning of no missing data, complete case analysis or no imputation applied, score Not applicable.* |  |
| **10a** | **Describe how predictors were handled in the analyses.** |  |
| i | For continuous predictors it is described whether they were modelled as linear, nonlinear (type of transformation specified) or categorized *A general statement is sufficient, no need to describe this for each predictor separately.  If no continuous predictors were reported, score Not applicable.* |  |
| ii | For categorical or categorized predictors, the cut-points were reported *If no categorical or categorized predictors were reported, score Not applicable.* |  |
| iii | For categorized predictors the method to choose the cut-points was clearly described *If no categorized predictors, score Not applicable.* |  |
| **10b** | **Specify type of model, all model-building procedures (including any predictor selection), and method for internal validation.** |  |
| i | The type of statistical model is reported *E.g. Logistic, Cox, other regression model (e.g. Weibull, ordinal), other statistical modelling (e.g. neural network)* |  |
| ii | The approach used for predictor selection before modelling is described *‘Before modelling’ means before any univariable or multivariable analysis of predictor-outcome associations. If no predictor selection before modelling is done, score Not applicable. If it is unclear whether predictor selection before modelling is done, score No. If it is clear there was predictor selection before modelling but the method was not described, score No.* |  |
| iii | The approach used for predictor selection during modelling is described *E.g. Univariable analysis, stepwise selection, bootstrap, Lasso. ‘During modelling’ includes both univariable or multivariable analysis of predictor-outcome associations.  If no predictor selection during modelling is done (so-called full model approach), score Not applicable. If it is unclear whether predictor selection during modelling is done, score No.  If it is clear there was predictor selection during modelling but the method was not described, score No.* |  |
| iv | Testing of interaction terms is described *If it is explicitly mentioned that interaction terms were not addressed in the prediction model, score Yes.  If interaction terms were included in the prediction model, but the testing is not described, score No.* |  |
| v | Testing of the proportionality of hazards in survival models is described *If no proportional hazard model is used, score Not applicable.* |  |
| vi | Internal validation is reported  *E.g. Bootstrapping, cross validation, split sample. If the use of internal validation is clearly a non-issue (e.g. in case of very large data sets), score Yes. For all other situations an explicit mention is expected.* |  |
| **10c** | **For validation, describe how the predictions were calculated.** |  |
| i. | It is described how predictions for individuals (in the validation set) were obtained from the model being validated  *E.g. Using the original reported model coefficients with or without the intercept, and/or using updated or refitted model coefficients, or using a nomogram, spreadsheet or web calculator.* | Not applicable |
| **10d** | **Specify all measures used to assess model performance and, if relevant, to compare multiple models.** *These should be described in methods section of the paper (item 16 addresses the reporting of the results for model performance).* |  |
| i | Measures for model discrimination are described *E.g. C-index / area under the ROC curve.* |  |
| ii | Measures for model calibration are described *E.g. calibration plot, calibration slope or intercept, calibration table, Hosmer Lemeshow test, O/E ratio*. |  |
| iii | Other performance measures are described  *E.g. R2, Brier score, predictive values, sensitivity, specificity, AUC difference, decision curve analysis, net reclassification improvement, integrated discrimination improvement, AIC.* |  |
| **10e** | **Describe any model updating (e.g., recalibration) arising from the validation, if done.** |  |
| i | A description of model-updating is given *E.g. Intercept recalibration, regression coefficient recalibration, refitting the whole model, adding a new predictor  If updating was done, it should be clear which updating method was applied to score Yes.  If it is not explicitly mentioned that updating was applied in the study, score this item as ‘Not applicable’.* | Not applicable |
| **11** | **Provide details on how risk groups were created, if done.** *If risk groups were not created, score this item as Yes.* |  |
| i | If risk groups were created, risk group boundaries (risk thresholds) are specified  *Score this item separately for development and validation if a study includes both development and validation. If risk groups were not created, score this item as not applicable.* |  |
| **12** | **For validation, identify any differences from the development data in setting, eligibility criteria, outcome and predictors.** |  |
| i | Differences or similarities in definitions with the development study are described *Mentioning of any differences in all four (setting, eligibility criteria, predictors and outcome) is required to score Yes.  If it is explicitly mentioned that there were no differences in setting, eligibility criteria, predictors and outcomes, score Yes.* | Not applicable |
| **Results** | |  |
| **13a** | **Describe the flow of participants through the study, including the number of participants with and without the outcome and, if applicable, a summary of the follow-up time. A diagram may be helpful.** |  |
| i | The flow of participants is reported |  |
| ii | The number of participants with and without the outcome are reported *If outcomes are continuous, score Not applicable.* |  |
| iii | A summary of follow-up time is presented *This notably applies to prognosis studies and diagnostic studies with follow-up as diagnostic outcome. If this is not applicable for an article (i.e. diagnostic study or no follow-up), then score Not applicable.* |  |
| **13b** | **Describe the characteristics of the participants (basic demographics, clinical features, available predictors), including the number of participants with missing data for predictors and outcome.** |  |
| i | Basic demographics are reported |  |
| ii | Summary information is provided for all predictors included in the final developed/validated model |  |
| iii | The number of participants with missing data for predictors is reported |  |
| iv | The number of participants with missing data for the outcome is reported |  |
| **13c** | **For validation, show a comparison with the development data of the distribution of important variables (demographics, predictors and outcome).** |  |
| i | Demographic characteristics (at least age and gender) of the validation study participants are reported along with those of the original development study | Not applicable |
| ii | Distributions of predictors in the model of the validation study participants are reported along with those of the original development study | Not applicable |
| iii | Outcomes of the validation study participants are reported along with those of the original development study | Not applicable |
| **14a** | **Specify the number of participants and outcome events in each analysis.** |  |
| i | The number of participants in each analysis (e.g. in the analysis of each model if more than one model is developed) is specified |  |
| ii | The number of outcome events in each analysis is specified (e.g. in the analysis of each model if more than one model is developed) *If outcomes are continuous, score Not applicable.* |  |
| **14b** | **If done, report the unadjusted association between each candidate predictor and outcome.** |  |
| i | The unadjusted associations between each predictor and outcome are reported *If any univariable analysis is mentioned in the methods but not in the results, score No.  If nothing on univariable analysis (in methods or results) is reported, score this item as Not applicable.* |  |
| **15a** | **Present the full prediction model to allow predictions for individuals (i.e., all regression coefficients, and model intercept or baseline survival at a given time point).** |  |
| i | The regression coefficient (or a derivative such as hazard ratio, odds ratio, risk ratio) for each predictor in the model is reported |  |
| ii | The intercept or the cumulative baseline hazard (or baseline survival) for at least one time point is reported |  |
| **15b** | **Explain how to use the prediction model.** |  |
| i | An explanation (e.g. a simplified scoring rule, chart, nomogram of the model, reference to online calculator, or worked example) is provided to explain how to use the model for individualised predictions. |  |
| **16** | **Report performance measures (with confidence intervals) for the prediction model.** *These should be described in results section of the paper (item 10 addresses the reporting of the methods for model performance).* |  |
| i | A discrimination measure is presented *E.g. C-index / area under the ROC curve.* |  |
| ii | The confidence interval (or standard error) of the discrimination measure is presented |  |
| iii | Measures for model calibration are described *E.g. calibration plot, calibration slope or intercept, calibration table, Hosmer Lemeshow test, O/E ratio.* |  |
| iv | Other model performance measures are presented *E.g. R2, Brier score, predictive values, sensitivity, specificity, AUC difference, decision curve analysis, net reclassification improvement, integrated discrimination improvement, AIC.* |  |
| **17** | **If done, report the results from any model updating (i.e., model specification, model performance, recalibration).** *If updating was not done, score this TRIPOD item as ‘Not applicable’.* |  |
| 0 | Model updating was done *If "No", then answer 17i-17v with "Not applicable"* | Not applicable |
| i | The updated regression coefficients for each predictor in the model are reported  *If model updating was described as ‘not needed’, score Yes.* | Not applicable |
| ii | The updated intercept or cumulative baseline hazard or baseline survival (for at least one time point) is reported  *If model updating was described as ‘not needed’, score Yes.* | Not applicable |
| iii | The discrimination of the updated model is reported | Not applicable |
| iv | The confidence interval (or standard error) of the discrimination measure of the updated model is reported | Not applicable |
| v | The calibration of the updated model is reported | Not applicable |
| **Discussion** | |  |
| **18** | **Discuss any limitations of the study (such as nonrepresentative sample, few events per predictor, missing data).** |  |
| i | Limitations of the study are discussed *Stating any limitation is sufficient.* |  |
| **19a** | **For validation, discuss the results with reference to performance in the development data, and any other validation data.** |  |
| i | Comparison of results to reported performance in development studies and/or other validation studies is given | Not applicable |
| **19b** | **Give an overall interpretation of the results considering objectives, limitations, results from similar studies and other relevant evidence.** |  |
| i | An overall interpretation of the results is given |  |
| **20** | **Discuss the potential clinical use of the model and implications for future research.** |  |
| i | The potential clinical use is discussed  *E.g. an explicit description of the context in which the prediction model is to be used (e.g. to identify high risk groups to help direct treatment, or to triage patients for referral to subsequent care).* |  |
| ii | Implications for future research are discussed *E.g. a description of what the next stage of investigation of the prediction model should be, such as ”We suggest further external validation”.* |  |
| **Other information** | |  |
| **21** | **Provide information about the availability of supplementary resources, such as study protocol, web calculator, and data sets.** |  |
| i | Information about supplementary resources is provided |  |
| **22** | **Give the source of funding and the role of the funders for the present study.** |  |
| i | The source of funding is reported or there is explicit mention that there was no external funding involved |  |
| ii | The role of funders is reported or there is explicit mention that there was no external funding |  |

**Supplementary information 3:**

References for the 140 studies describing prognostic models, included in the systematic review.

1. Xiao Y, Zheng X, Zhou C, et al (2022) Combined hepatocellular carcinoma-cholangiocarcinoma with a predominant HCC component: better survival and MRI-based prediction. Eur Radiol 33:1412–1421. https://doi.org/10.1007/s00330-022-09131-5
2. Bian Y, Zheng Z, Fang X, et al (2023) Artificial Intelligence to Predict Lymph Node Metastasis at CT in Pancreatic Ductal Adenocarcinoma. Radiology 306:160–169. https://doi.org/10.1148/radiol.220329
3. Feng Z, Li H, Liu Q, et al (2023) CT Radiomics to Predict Macrotrabecular-Massive Subtype and Immune Status in Hepatocellular Carcinoma. Radiology 307:e221291. https://doi.org/10.1148/radiol.221291
4. Kitao A, Matsui O, Zhang Y, et al (2023) Dynamic CT and Gadoxetic Acid-enhanced MRI Characteristics of P53-mutated Hepatocellular Carcinoma. Radiology 306:e220531. https://doi.org/10.1148/radiol.220531
5. Yeom A, Chi SA, Song KD (2023) Added Value of Pelvic CT after Treatment of HCC. Radiology 307:e222314. https://doi.org/10.1148/radiol.222314
6. Jiang H, Wei H, Yang T, et al (2023) VICT2 Trait: Prognostic Alternative to Peritumoral Hepatobiliary Phase Hypointensity in HCC. Radiology 307:e221835. https://doi.org/10.1148/radiol.221835
7. Lin Y-M, Paolucci I, O’Connor CS, et al (2023) Ablative Margins of Colorectal Liver Metastases Using Deformable CT Image Registration and Autosegmentation. Radiology 307:e221373. https://doi.org/10.1148/radiol.221373
8. Peng Y, Shen H, Tang H, et al (2022) Nomogram based on CT–derived extracellular volume for the prediction of post-hepatectomy liver failure in patients with resectable hepatocellular carcinoma. Eur Radiol 32:8529–8539. https://doi.org/10.1007/s00330-022-08917-x
9. Seo N, Joo DJ, Park M-S, et al (2022) Optimal imaging criteria and modality to determine Milan criteria for the prediction of post-transplant HCC recurrence after locoregional treatment. Eur Radiol 33:501–511. https://doi.org/10.1007/s00330-022-08977-z
10. Tan B, Tang Z, Ou J, et al (2022) A novel model based on liver/spleen volumes and portal vein diameter on MRI to predict variceal bleeding in HBV cirrhosis. Eur Radiol 33:1378–1387. https://doi.org/10.1007/s00330-022-09107-5
11. Xia T, Zhou Z, Meng X, et al (2023) Predicting Microvascular Invasion in Hepatocellular Carcinoma Using CT-based Radiomics Model. Radiology 307:e222729. https://doi.org/10.1148/radiol.222729
12. Gou X, Jia W, He C, et al (2022) Hepatic hydrothorax does not increase the risk of death after transjugular intrahepatic portosystemic shunt in cirrhosis patients. Eur Radiol 33:3407–3415. https://doi.org/10.1007/s00330-022-09357-3
13. Tada A, Nagai T, Kato Y, et al (2022) Liver stiffness assessed by magnetic resonance elastography predicts clinical outcomes in patients with heart failure and without chronic liver disease. Eur Radiol 33:2062–2074. https://doi.org/10.1007/s00330-022-09209-0
14. Zhao Q, He X, Wang K, et al (2022) Deep learning model based on contrast-enhanced ultrasound for predicting early recurrence after thermal ablation of colorectal cancer liver metastasis. Eur Radiol 33:1895–1905. https://doi.org/10.1007/s00330-022-09203-6
15. Cai S, Lin N, Yang Y, et al (2023) The value of contrast-enhanced portal vein imaging at the hepatobiliary phase obtained with gadobenate dimeglumine for predicting decompensation and transplant-free survival in chronic liver disease. Eur Radiol 33:3425–3434. https://doi.org/10.1007/s00330-023-09489-0
16. Chu HH, Gwon DI, Kim GH, et al (2022) Balloon-occluded transarterial chemoembolization versus conventional transarterial chemoembolization for the treatment of single hepatocellular carcinoma: a propensity score matching analysis. Eur Radiol 33:2655–2664. https://doi.org/10.1007/s00330-022-09284-3
17. Wu F, Sun H, Zhou C, et al (2023) Prognostic factors for long-term outcome in bifocal hepatocellular carcinoma after resection. Eur Radiol 33:3604–3616. https://doi.org/10.1007/s00330-023-09398-2
18. Lee HS, Kwon HW, Lim S-B, et al (2022) FDG metabolic parameter-based models for predicting recurrence after upfront surgery in synchronous colorectal cancer liver metastasis. Eur Radiol 33:1746–1756. https://doi.org/10.1007/s00330-022-09141-3
19. Liu K, Zheng H, Sui X, et al (2022) Microwave ablation versus surgical resection for subcapsular hepatocellular carcinoma: a propensity score–matched study of long-term therapeutic outcomes. Eur Radiol 33:1938–1948. https://doi.org/10.1007/s00330-022-09135-1
20. Park SH, Kim B, Kim S, et al (2023) Estimating postsurgical outcomes of patients with a single hepatocellular carcinoma using gadoxetic acid–enhanced MRI: risk scoring system development and validation. Eur Radiol 33:3566–3579. https://doi.org/10.1007/s00330-023-09539-7
21. Cha H, Choi J-Y, Park YN, et al (2022) Comparison of imaging findings of macrotrabecular-massive hepatocellular carcinoma using CT and gadoxetic acid–enhanced MRI. Eur Radiol 33:1364–1377. https://doi.org/10.1007/s00330-022-09105-7
22. Jang EB, Kim DW, Choi SH, et al (2022) Transient severe motion artifacts on gadoxetic acid–enhanced MRI: risk factor analysis in 2230 patients. Eur Radiol 32:8629–8638. https://doi.org/10.1007/s00330-022-08885-2
23. Meng D, Liang C, Zheng Y, et al (2022) The value of gadobenate dimeglumine–enhanced biliary imaging from the hepatobiliary phase for predicting post-hepatectomy liver failure in HCC patients. Eur Radiol 32:8608–8616. https://doi.org/10.1007/s00330-022-08874-5
24. Chen B-B, Liang P-C, Shih TT-F, et al (2022) Sarcopenia and myosteatosis are associated with survival in patients receiving immunotherapy for advanced hepatocellular carcinoma. Eur Radiol 33:512–522. https://doi.org/10.1007/s00330-022-08980-4
25. Jiao CY, Zhang H, Ji GW, et al (2022) CT-based clinico-radiological nomograms for prognosis prediction in patients with intrahepatic mass-forming cholangiocarcinoma: a multi-institutional study. Eur Radiol 32:8326–8338. https://doi.org/10.1007/s00330-022-08914-0
26. Öcal O, Zech CJ, Fabritius MP, et al (2022) Non-hypervascular hepatobiliary phase hypointense lesions detected in patients with hepatocellular carcinoma: a post hoc analysis of SORAMIC trial to identify risk factors for progression. Eur Radiol 33:493–500. https://doi.org/10.1007/s00330-022-09000-1
27. Müller L, Mähringer-Kunz A, Auer TA, et al (2022) Low bone mineral density is a prognostic factor for elderly patients with HCC undergoing TACE: results from a multicenter study. Eur Radiol 33:1031–1039. https://doi.org/10.1007/s00330-022-09069-8
28. Park HJ, Kim HJ, Kim JH, et al (2022) Prognostic value of tumor-to-parenchymal contrast enhancement ratio on portal venous-phase CT in pancreatic neuroendocrine neoplasms. Eur Radiol 33:2713–2724. https://doi.org/10.1007/s00330-022-09235-y
29. Wu T, Yang D, Wee A, et al (2022) Identification of MRI features associated with injury type, severity, and prognosis in drug-induced liver injury. Eur Radiol 33:666–677. https://doi.org/10.1007/s00330-022-09041-6
30. Chen Y, Huang W, Liu Q, et al (2022) Covered stent treatment for arterial complications after pancreatic surgery: risk assessment for recurrence and peri-stent implantation management. Eur Radiol 33:1779–1791. https://doi.org/10.1007/s00330-022-09134-2
31. Maruzzelli L, D’Amico M, Tuzzolino F, et al (2022) Clinical outcomes of transjugular intrahepatic portosystemic shunt with PTFE-covered stents after liver transplantation and technical results in split and whole liver graft recipients. Eur Radiol 33:2612–2619. https://doi.org/10.1007/s00330-022-09259-4
32. Fan W, Zhu B, Yue S, et al (2022) Identifying optimal candidates for post-TIPS patients with HCC undergoing TACE: a multicenter observational study. Eur Radiol 33:2809–2820. https://doi.org/10.1007/s00330-022-09249-6
33. Wang L, Feng B, Li D, et al (2023) Risk stratification of solitary hepatocellular carcinoma ≤ 5 cm without microvascular invasion: prognostic values of MR imaging features based on LI-RADS and clinical parameters. Eur Radiol 33:3592–3603. https://doi.org/10.1007/s00330-023-09484-5
34. Kolossváry M, Raghu VK, Nagurney JT, et al (2023) Deep Learning Analysis of Chest Radiographs to Triage Patients with Acute Chest Pain Syndrome. Radiology 306:e221926. https://doi.org/10.1148/radiol.221926
35. Chen Q, Shao J, Xue T, et al (2022) Intratumoral and peritumoral radiomics nomograms for the preoperative prediction of lymphovascular invasion and overall survival in non-small cell lung cancer. Eur Radiol 33:947–958. https://doi.org/10.1007/s00330-022-09109-3
36. Zhang X, Lu B, Yang X, et al (2022) Prognostic analysis and risk stratification of lung adenocarcinoma undergoing EGFR-TKI therapy with time-serial CT-based radiomics signature. Eur Radiol 33:825–835. https://doi.org/10.1007/s00330-022-09123-5
37. Hahn AD, Carey KJ, Barton GP, et al (2022) Hyperpolarized 129Xe MR Spectroscopy in the Lung Shows 1-year Reduced Function in Idiopathic Pulmonary Fibrosis. Radiology 305:688–696. https://doi.org/10.1148/radiol.211433
38. Bhatt SP, Bodduluri S, Nakhmani A, et al (2022) Sex Differences in Airways at Chest CT: Results from the COPDGene Cohort. Radiology 305:699–708. https://doi.org/10.1148/radiol.212985
39. Lee JE, Chae KJ, Suh YJ, et al (2023) Prevalence and Long-term Outcomes of CT Interstitial Lung Abnormalities in a Health Screening Cohort. Radiology 306:e221172. https://doi.org/10.1148/radiol.221172
40. Tsakok MT, Watson RA, Saujani SJ, et al (2023) Reduction in Chest CT Severity and Improved Hospital Outcomes in SARS-CoV-2 Omicron Compared with Delta Variant Infection. Radiology 306:261–269. https://doi.org/10.1148/radiol.220533
41. Díaz AA, Nardelli P, Wang W, et al (2023) Artificial Intelligence-based CT Assessment of Bronchiectasis: The COPDGene Study. Radiology 307:e221109. https://doi.org/10.1148/radiol.221109
42. Kim H, Jin KN, Yoo S-J, et al (2023) Deep Learning for Estimating Lung Capacity on Chest Radiographs Predicts Survival in Idiopathic Pulmonary Fibrosis. Radiology 306:e220292. https://doi.org/10.1148/radiol.220292
43. Baraghoshi D, Strand M, Humphries SM, et al (2023) Quantitative CT Evaluation of Emphysema Progression over 10 Years in the COPDGene Study. Radiology 307:e222786. https://doi.org/10.1148/radiol.222786
44. Park S-J, Lee S, Lee HN, Cho Y (2022) Early versus delayed bronchial artery embolization for non-massive hemoptysis. Eur Radiol 33:116–124. https://doi.org/10.1007/s00330-022-08993-z
45. Li Y, Wang B, Wen L, et al (2022) Machine learning and radiomics for the prediction of multidrug resistance in cavitary pulmonary tuberculosis: a multicentre study. Eur Radiol 33:391–400. https://doi.org/10.1007/s00330-022-08997-9
46. Nam JG, Choi Y, Lee S-M, et al (2023) Prognostic value of deep learning–based fibrosis quantification on chest CT in idiopathic pulmonary fibrosis. Eur Radiol 33:3144–3155. https://doi.org/10.1007/s00330-023-09534-y
47. Li Y, Liu J, Yang X, et al (2023) An ordinal radiomic model to predict the differentiation grade of invasive non-mucinous pulmonary adenocarcinoma based on low-dose computed tomography in lung cancer screening. Eur Radiol 33:3072–3082. https://doi.org/10.1007/s00330-023-09453-y
48. Wrobel MM, Cahalane AM, Pachamanova D, et al (2022) Comparison of expected imaging findings following percutaneous microwave and cryoablation of pulmonary tumors: ablation zones and thoracic lymph nodes. Eur Radiol 32:8171–8181. https://doi.org/10.1007/s00330-022-08905-1
49. Mascalchi M, Romei C, Marzi C, et al (2023) Pulmonary emphysema and coronary artery calcifications at baseline LDCT and long-term mortality in smokers and former smokers of the ITALUNG screening trial. Eur Radiol 33:3115–3123. https://doi.org/10.1007/s00330-023-09504-4
50. Park D, Oh D, Lee M, et al (2022) Importance of CT image normalization in radiomics analysis: prediction of 3-year recurrence-free survival in non-small cell lung cancer. Eur Radiol 32:8716–8725. https://doi.org/10.1007/s00330-022-08869-2
51. Zhang L, Xu C, Zhang X, et al (2022) A novel analytical approach for outcome prediction in newly diagnosed NSCLC based on [18F]FDG PET/CT metabolic parameters, inflammatory markers, and clinical variables. Eur Radiol 33:1757–1768. https://doi.org/10.1007/s00330-022-09150-2
52. He L, Li Z-H, Yan L-X, et al (2022) Development and validation of a computed tomography–based immune ecosystem diversity index as an imaging biomarker in non-small cell lung cancer. Eur Radiol 32:8726–8736. https://doi.org/10.1007/s00330-022-08873-6
53. Oh S, Lee JW, Yoo S-Y, et al (2022) Pleuroparenchymal fibroelastosis after hematopoietic stem cell transplantation in children: a propensity score–matched analysis. Eur Radiol 33:2266–2276. https://doi.org/10.1007/s00330-022-09188-2
54. Lee JE, Hwang M, Kim Y-H, et al (2023) SARS-CoV-2 Variants Infection in Relationship to Imaging-based Pneumonia and Clinical Outcomes. Radiology 306:e221795. https://doi.org/10.1148/radiol.221795
55. Park S, Lee SM, Choe J, et al (2023) Recurrence Patterns and Patient Outcomes in Resected Lung Adenocarcinoma Differ according to Ground-Glass Opacity at CT. Radiology 307:e222422. https://doi.org/10.1148/radiol.222422
56. Chen Q, Pan T, Wang YN, et al (2023) A Coronary CT Angiography Radiomics Model to Identify Vulnerable Plaque and Predict Cardiovascular Events. Radiology 307:e221693. https://doi.org/10.1148/radiol.221693
57. Li Y, Xu Y, Li W, et al (2023) Cardiac MRI to Predict Sudden Cardiac Death Risk in Dilated Cardiomyopathy. Radiology 307:e222552. https://doi.org/10.1148/radiol.222552
58. Yang S, Hoshino M, Koo B-K, et al (2022) Relationship of Plaque Features at Coronary CT to Coronary Hemodynamics and Cardiovascular Events. Radiology 305:578–587. https://doi.org/10.1148/radiol.213271
59. Ghanbari F, Joyce T, Lorenzoni V, et al (2023) AI Cardiac MRI Scar Analysis Aids Prediction of Major Arrhythmic Events in the Multicenter DERIVATE Registry. Radiology 307:e222239. https://doi.org/10.1148/radiol.222239
60. Yu M-M, Tang X-L, Zhao X, et al (2022) Plaque progression at coronary CT angiography links non-alcoholic fatty liver disease and cardiovascular events: a prospective single-center study. Eur Radiol 32:8111–8121. https://doi.org/10.1007/s00330-022-08904-2
61. Yu Y, Ding X, Yu L, et al (2022) Prediction of microvascular complications in diabetic patients without obstructive coronary stenosis based on peri-coronary adipose tissue attenuation model. Eur Radiol 33:2015–2026. https://doi.org/10.1007/s00330-022-09176-6
62. Jiang J, Wei J, Zhu Y, et al (2022) Clot-based radiomics model for cardioembolic stroke prediction with CT imaging before recanalization: a multicenter study. Eur Radiol 33:970–980. https://doi.org/10.1007/s00330-022-09116-4
63. Pamminger M, Reindl M, Kranewitter C, et al (2022) Prognostic value of pulmonary transit time by cardiac magnetic resonance imaging in ST-elevation myocardial infarction. Eur Radiol 33:1219–1228. https://doi.org/10.1007/s00330-022-09050-5
64. Pu L, Diao Y, Wang J, et al (2022) The predictive value of fast semi-automated left atrial long-axis strain analysis for atrial fibrillation in hypertrophic cardiomyopathy. Eur Radiol 33:312–320. https://doi.org/10.1007/s00330-022-09020-x
65. Yu M-M, Zuo W-X, Zhao X, et al (2023) Prognostic value of coronary CT angiography in heart failure patients with preserved ejection fraction. Eur Radiol 33:3052–3063. https://doi.org/10.1007/s00330-022-09380-4
66. Chen B-H, Wu C-W, An D-A, et al (2022) Myocardial extracellular volume quantified by cardiac magnetic resonance predicts left ventricular aneurysm following acute myocardial infarction. Eur Radiol 33:283–293. https://doi.org/10.1007/s00330-022-08995-x
67. Jiang L, Guo Y-K, Xu H-Y, et al (2022) Incremental prognostic value of myocardial strain over ventricular volume in patients with repaired tetralogy of Fallot. Eur Radiol 33:1992–2003. https://doi.org/10.1007/s00330-022-09166-8
68. Usami K, Watabe H, Hoshi T, et al (2022) Impact of coronary plaque characteristics on periprocedural myocardial injury in elective percutaneous coronary intervention. Eur Radiol 33:3020–3028. https://doi.org/10.1007/s00330-022-09288-z
69. Zu ZY, Xu PP, Chen Q, et al (2022) The prognostic value of CT-derived fractional flow reserve in coronary artery bypass graft: a retrospective multicenter study. Eur Radiol 33:3029–3040. https://doi.org/10.1007/s00330-022-09353-7
70. Xi Y, Huang L, Hao J, et al (2022) Predictive performance of the perivascular fat attenuation index for interventional antegrade percutaneous coronary intervention for chronic total occlusion. Eur Radiol 33:3041–3051. https://doi.org/10.1007/s00330-022-09370-6
71. Wang L, Wang Y, Wang J, et al (2023) Myocardial Activity at 18F-FAPI PET/CT and Risk for Sudden Cardiac Death in Hypertrophic Cardiomyopathy. Radiology 306:e221052. https://doi.org/10.1148/radiol.221052
72. Li S, Wang Y, Yang W, et al (2023) Cardiac MRI Risk Stratification for Dilated Cardiomyopathy with Left Ventricular Ejection Fraction of 35% or Higher. Radiology 306:e213059. https://doi.org/10.1148/radiol.213059
73. Mauger CA, Gilbert K, Suinesiaputra A, et al (2023) Multi-Ethnic Study of Atherosclerosis: Relationship between Left Ventricular Shape at Cardiac MRI and 10-year Outcomes. Radiology 306:e220122. https://doi.org/10.1148/radiol.220122
74. Figliozzi S, Georgiopoulos G, Lopes PM, et al (2023) Myocardial Fibrosis at Cardiac MRI Helps Predict Adverse Clinical Outcome in Patients with Mitral Valve Prolapse. Radiology 306:112–121. https://doi.org/10.1148/radiol.220454
75. Fukutomi H, Yamamoto T, Sibon I, et al (2023) Location-weighted versus Volume-weighted Mismatch at MRI for Response to Mechanical Thrombectomy in Acute Stroke. Radiology 306:e220080. https://doi.org/10.1148/radiol.220080
76. Hou Z, Jing J, Yan L, et al (2023) New Diffusion Abnormalities Following Endovascular Treatment for Intracranial Atherosclerosis. Radiology 307:e221499. https://doi.org/10.1148/radiol.221499
77. Abdelrady M, Derraz I, Lefevre P-H, et al (2022) Negative susceptibility vessel sign might be predictive of complete reperfusion in patients with acute basilar artery occlusion managed with thrombectomy. Eur Radiol 33:2593–2604. https://doi.org/10.1007/s00330-022-09215-2
78. Wang Z-M, Wei P-H, Wang C, et al (2022) Combined [18F]FDG-PET with MRI structural patterns in predicting post-surgical seizure outcomes in temporal lobe epilepsy patients. Eur Radiol 32:8423–8431. https://doi.org/10.1007/s00330-022-08912-2
79. Xu X-Q, Ma G, Lu S-S, et al (2022) Predictors of ghost infarct core on baseline computed tomography perfusion in stroke patients with successful recanalization after mechanical thrombectomy. Eur Radiol 33:1792–1800. https://doi.org/10.1007/s00330-022-09189-1
80. Xu X-Q, Shen G-C, Ma G, et al (2022) Prognostic value of post-treatment fluid-attenuated inversion recovery vascular hyperintensity in ischemic stroke after endovascular thrombectomy. Eur Radiol 32:8067–8076. https://doi.org/10.1007/s00330-022-08886-1
81. Rozenblum L, Zaragori T, Tran S, et al (2022) Differentiating high-grade glioma progression from treatment-related changes with dynamic [18F]FDOPA PET: a multicentric study. Eur Radiol 33:2548–2560. https://doi.org/10.1007/s00330-022-09221-4
82. Song JH, Park D, Jeong Y-S, Park JK (2022) Potential role of bolus-tracking data of carotid CT angiography for atrial fibrillation prediction. Eur Radiol 33:981–987. https://doi.org/10.1007/s00330-022-09072-z
83. Xing P, Zhou X, Shen F, et al (2022) Imaging mismatch between Alberta Stroke Program Early CT Score and perfusion imaging may be a good variable for endovascular treatment. Eur Radiol 33:2629–2637. https://doi.org/10.1007/s00330-022-09273-6
84. Xu X, Zhang G, Jia Z, et al (2022) Predictors of malignant middle cerebral artery infarction after endovascular thrombectomy: results of DIRECT-MT trial. Eur Radiol 33:135–143. https://doi.org/10.1007/s00330-022-09013-w
85. Lu M, Zhang H, Liu D, et al (2022) Vessel wall enhancement as a predictor of arterial stenosis progression and poor outcomes in moyamoya disease. Eur Radiol 33:2489–2499. https://doi.org/10.1007/s00330-022-09223-2
86. Park YW, Kim S, Park CJ, et al (2022) Adding radiomics to the 2021 WHO updates may improve prognostic prediction for current IDH-wildtype histological lower-grade gliomas with known EGFR amplification and TERT promoter mutation status. Eur Radiol 32:8089–8098. https://doi.org/10.1007/s00330-022-08941-x
87. Wyss PO, Richter JK, Zweers P, et al (2023) Glutathione in the Pons Is Associated With Clinical Status Improvements in Subacute Spinal Cord Injury. Invest Radiol 58:131–138. https://doi.org/10.1097/RLI.0000000000000905
88. Xu X-Q, Chu Y, Shen G-C, et al (2022) Prognostic value of ASPECTS on post-treatment diffusion-weighted imaging for acute ischemic stroke patients after endovascular thrombectomy: comparison with infarction volume. Eur Radiol 32:8079–8088. https://doi.org/10.1007/s00330-022-08888-z
89. Keijzer HM, Duering M, Pasternak O, et al (2022) Free water corrected diffusion tensor imaging discriminates between good and poor outcomes of comatose patients after cardiac arrest. Eur Radiol 33:2139–2148. https://doi.org/10.1007/s00330-022-09245-w
90. Liu D, Chen J, Ge H, et al (2022) Radiogenomics to characterize the immune-related prognostic signature associated with biological functions in glioblastoma. Eur Radiol 33:209–220. https://doi.org/10.1007/s00330-022-09012-x
91. Rutten C, Fillon L, Kuchenbuch M, et al (2022) The longitudinal evolution of cerebral blood flow in children with tuberous sclerosis assessed by arterial spin labeling magnetic resonance imaging may be related to cognitive performance. Eur Radiol 33:196–206. https://doi.org/10.1007/s00330-022-09036-3
92. Salem MM, Kuybu O, Nguyen Hoang A, et al (2023) Middle Meningeal Artery Embolization for Chronic Subdural Hematoma: Predictors of Clinical and Radiographic Failure from 636 Embolizations. Radiology 307:e222045. https://doi.org/10.1148/radiol.222045
93. Morotti A, Busto G, Boulouis G, et al (2022) Added value of non-contrast CT and CT perfusion markers for prediction of intracerebral hemorrhage expansion and outcome. Eur Radiol 33:690–698. https://doi.org/10.1007/s00330-022-08987-x
94. Lee SH, Jang M-J, Yoen H, et al (2023) Background Parenchymal Enhancement at Postoperative Surveillance Breast MRI: Association with Future Second Breast Cancer Risk. Radiology 306:90–99. https://doi.org/10.1148/radiol.220440
95. Tran TXM, Kim S, Song H, et al (2023) Association of Longitudinal Mammographic Breast Density Changes with Subsequent Breast Cancer Risk. Radiology 306:e220291. https://doi.org/10.1148/radiol.220291
96. Ramtohul T, Tescher C, Vaflard P, et al (2022) Prospective Evaluation of Ultrafast Breast MRI for Predicting Pathologic Response after Neoadjuvant Therapies. Radiology 305:565–574. https://doi.org/10.1148/radiol.220389
97. Loving VA, Johnston BS, Reddy DH, et al (2023) Antithrombotic Therapy and Hematoma Risk during Image-guided Core-Needle Breast Biopsy. Radiology 306:79–86. https://doi.org/10.1148/radiol.220548
98. Hwangbo L, Kim JY, Kim JJ, et al (2022) Changes in kinetic heterogeneity of breast cancer via computer-aided diagnosis on MRI predict the pathological response to neoadjuvant systemic therapy. Eur Radiol 33:440–449. https://doi.org/10.1007/s00330-022-08998-8
99. Park AR, Chae EY, Kim HJ, et al (2023) Effects of Preoperative Breast MRI on Breast Cancer Survival Outcomes in Women Aged 35 Years and Younger. Radiology 307:e221797. https://doi.org/10.1148/radiol.221797
100. Ragusi MAA, Van Der Velden BHM, Meeuwis C, et al (2023) Long-term Survival in Breast Cancer Patients Is Associated with Contralateral Parenchymal Enhancement at MRI: Outcomes of the SELECT Study. Radiology 307:e221922. https://doi.org/10.1148/radiol.221922
101. Fong W, Tan L, Tan C, et al (2022) Predicting the risk of axillary lymph node metastasis in early breast cancer patients based on ultrasonographic-clinicopathologic features and the use of nomograms: a prospective single-center observational study. Eur Radiol 32:8200–8212. https://doi.org/10.1007/s00330-022-08855-8
102. Goh Y, Chou C-P, Chan CW, et al (2022) Impact of contrast-enhanced mammography in surgical management of breast cancers for women with dense breasts: a dual-center, multi-disciplinary study in Asia. Eur Radiol 32:8226–8237. https://doi.org/10.1007/s00330-022-08906-0
103. Xu Z, Ding Y, Zhao K, et al (2022) MRI characteristics of breast edema for assessing axillary lymph node burden in early-stage breast cancer: a retrospective bicentric study. Eur Radiol 32:8213–8225. https://doi.org/10.1007/s00330-022-08896-z
104. D’Amore S, Sano H, Chappell DDG, et al (2023) Radiographic Cortical Thickness Index Predicts Fragility Fracture in Gaucher Disease. Radiology 307:e212779. https://doi.org/10.1148/radiol.212779
105. Napoli A, Alfieri G, De Maio A, et al (2023) CT-guided Pulsed Radiofrequency Combined with Steroid Injection for Sciatica from Herniated Disk: A Randomized Trial. Radiology 307:e221478. https://doi.org/10.1148/radiol.221478
106. Tschopp M, Pfirrmann CWA, Fucentese SF, et al (2023) A Randomized Trial of Intra-articular Injection Therapy for Knee Osteoarthritis. Invest Radiol 58:355–362. https://doi.org/10.1097/RLI.0000000000000942
107. Janacova V, Szomolanyi P, Kirner A, et al (2022) Adjacent cartilage tissue structure after successful transplantation: a quantitative MRI study using T2 mapping and texture analysis. Eur Radiol 32:8364–8375. https://doi.org/10.1007/s00330-022-08897-y
108. Mohajer B, Moradi K, Guermazi A, et al (2022) Diabetes-associated thigh muscle degeneration mediates knee osteoarthritis–related outcomes: results from a longitudinal cohort study. Eur Radiol 33:595–605. https://doi.org/10.1007/s00330-022-09035-4
109. Tordjman M, Honoré C, Crombé A, et al (2022) Prognostic factors of the synovial sarcoma of the extremities: imaging does matter. Eur Radiol 33:1162–1173. https://doi.org/10.1007/s00330-022-09049-y
110. Li J, Zhang Z, Xie T, et al (2022) The preoperative Hounsfield unit value at the position of the future screw insertion is a better predictor of screw loosening than other methods. Eur Radiol 33:1526–1536. https://doi.org/10.1007/s00330-022-09157-9
111. Yin X, Tang N, Fan X, et al (2022) Mid-term efficacy grading evaluation and predictive factors of magnetic resonance–guided focused ultrasound surgery for painful bone metastases: a multi-center study. Eur Radiol 33:1465–1474. https://doi.org/10.1007/s00330-022-09118-2
112. Huang Y-C, Tsuang F-Y, Lee C-W, Lin Y-H (2022) Efficacy of preoperative embolization for metastatic spinal tumor surgery using angiographic vascularity assessment. Eur Radiol 33:2638–2646. https://doi.org/10.1007/s00330-022-09276-3
113. Bian L, Wu D, Chen Y, et al (2022) Associations of radiological features of adipose tissues with postoperative complications and overall survival of gastric cancer patients. Eur Radiol 32:8569–8578. https://doi.org/10.1007/s00330-022-08918-w
114. Chen X, He L, Li Q, et al (2022) Non-invasive prediction of microsatellite instability in colorectal cancer by a genetic algorithm–enhanced artificial neural network–based CT radiomics signature. Eur Radiol 33:11–22. https://doi.org/10.1007/s00330-022-08954-6
115. Yan S, Shi Y-J, Liu C, et al (2022) Quantitative CT evaluation after two cycles of induction chemotherapy to predict prognosis of patients with locally advanced oesophageal squamous cell carcinoma before undergoing definitive chemoradiotherapy/radiotherapy. Eur Radiol 33:380–390. https://doi.org/10.1007/s00330-022-08994-y
116. Hofmann FO, Heinemann V, D’Anastasi M, et al (2022) Standard diametric versus volumetric early tumor shrinkage as a predictor of survival in metastatic colorectal cancer: subgroup findings of the randomized, open-label phase III trial FIRE-3 / AIO KRK-0306. Eur Radiol 33:1174–1184. https://doi.org/10.1007/s00330-022-09053-2
117. Zhang G, Xu Z, Zheng J, et al (2022) Prognostic value of multi b-value DWI in patients with locally advanced rectal cancer. Eur Radiol 33:1928–1937. https://doi.org/10.1007/s00330-022-09159-7
118. Seo N, Lim JS, Chung T, et al (2022) Preoperative computed tomography assessment of circumferential resection margin in retroperitonealized colon cancer predicts disease-free survival. Eur Radiol 33:2757–2767. https://doi.org/10.1007/s00330-022-09222-3
119. Han X, Zhang Q, Zhou N, et al (2022) Combined ultrasonography and CT for prognosis and predicting clinical outcomes of patients with pseudomyxoma peritonei. Eur Radiol 33:2800–2808. https://doi.org/10.1007/s00330-022-09242-z
120. Moore JL, Subesinghe M, Santaolalla A, et al (2023) Metabolic tumour and nodal response to neoadjuvant chemotherapy on FDG PET-CT as a predictor of pathological response and survival in patients with oesophageal adenocarcinoma. Eur Radiol 33:3647–3659. https://doi.org/10.1007/s00330-023-09482-7
121. Liu Y, Peng C, Chai H, et al (2022) Predicting ultrasound-guided thermal ablation benefit in primary hyperparathyroidism. Eur Radiol 32:8497–8506. https://doi.org/10.1007/s00330-022-08898-x
122. Yan L, Li X, Li Y, et al (2022) Comparison of ultrasound-guided radiofrequency ablation versus thyroid lobectomy for T1bN0M0 papillary thyroid carcinoma. Eur Radiol 33:730–740. https://doi.org/10.1007/s00330-022-08963-5
123. Anzai Y, Chang C-P, Rowe K, et al (2023) Surveillance Imaging with PET/CT and CT and/or MRI for Head and Neck Cancer and Mortality: A Population-based Study. Radiology 307:e212915. https://doi.org/10.1148/radiol.212915
124. Zheng L, Dou J-P, Han Z-Y, et al (2023) Microwave Ablation for Papillary Thyroid Microcarcinoma with and without US-detected Capsule Invasion: A Multicenter Prospective Cohort Study. Radiology 307:e220661. https://doi.org/10.1148/radiol.220661
125. Dongxiang W, Liting L, Yujing L, et al (2022) Prediction of outcomes in patients with local recurrent nasopharyngeal carcinoma: development and validation of a four-factor prognostic model integrating baseline characteristics and [18F]FDG PET/CT parameters. Eur Radiol 33:2840–2849. https://doi.org/10.1007/s00330-022-09232-1
126. Jiang Y, Liang Z, Chen K, et al (2022) A dynamic nomogram combining tumor stage and magnetic resonance imaging features to predict the response to induction chemotherapy in locally advanced nasopharyngeal carcinoma. Eur Radiol 33:2171–2184. https://doi.org/10.1007/s00330-022-09201-8
127. Liu Y, Peng C, Chai H, et al (2022) Predicting ultrasound-guided thermal ablation benefit in primary hyperparathyroidism. Eur Radiol 32:8497–8506. https://doi.org/10.1007/s00330-022-08898-x
128. Sun X-S, Xiao Z-W, Liu S-L, et al (2023) Nasopharyngeal necrosis contributes to overall survival in nasopharyngeal carcinoma without distant metastasis: a comprehensive nomogram model. Eur Radiol 33:3682–3692. https://doi.org/10.1007/s00330-023-09431-4
129. Zhao X, Li W, Zhang J, et al (2022) Radiomics analysis of CT imaging improves preoperative prediction of cervical lymph node metastasis in laryngeal squamous cell carcinoma. Eur Radiol 33:1121–1131. https://doi.org/10.1007/s00330-022-09051-4
130. Seifert R, Rasul S, Seitzer K, et al (2023) A Prognostic Risk Score for Prostate Cancer Based on PSMA PET–derived Organ-specific Tumor Volumes. Radiology 307:e222010. https://doi.org/10.1148/radiol.222010
131. Aarts BM, Gomez FM, Lopez-Yurda M, et al (2022) Safety and efficacy of RFA versus MWA for T1a renal cell carcinoma: a propensity score analysis. Eur Radiol 33:1040–1049. https://doi.org/10.1007/s00330-022-09110-w
132. Park MY, Park KJ, Kim M-H, Kim JK (2022) Focal nodular enhancement on DCE MRI of the prostatectomy bed: radiologic-pathologic correlations and prognostic value. Eur Radiol 33:2985–2994. https://doi.org/10.1007/s00330-022-09241-0
133. Rud E, Noor D, Galtung KF, et al (2022) Validating the screening criteria for bone metastases in treatment-naïve unfavorable intermediate and high-risk prostate cancer - the prevalence and location of bone- and lymph node metastases. Eur Radiol 32:8266–8275. https://doi.org/10.1007/s00330-022-08945-7
134. Wang J, Dong P, Qu Y, et al (2022) Association of computed tomography-based body composition with survival in metastatic renal cancer patient received immunotherapy: a multicenter, retrospective study. Eur Radiol 33:3232–3242. https://doi.org/10.1007/s00330-022-09345-7
135. Lee MH, Zea R, Garrett JW, et al (2023) Abdominal CT Body Composition Thresholds Using Automated AI Tools for predicting 10-year Adverse Outcomes. Radiology 306:e220574. https://doi.org/10.1148/radiol.220574
136. Iravani A, Wallace R, Lo SN, et al (2023) FDG PET/CT Prognostic Markers in Patients with Advanced Melanoma treated with Ipilimumab and Nivolumab. Radiology 307:e221180. https://doi.org/10.1148/radiol.221180
137. Hu Y, Ma M, Yin H, et al (2022) Assessment of cumulative cancer risk attributable to diagnostic X-ray radiation: a large cohort study. Eur Radiol 33:1769–1778. https://doi.org/10.1007/s00330-022-09178-4
138. Li M, Yao H, Zhang P, et al (2022) Development and validation of a [18F]FDG PET/CT-based radiomics nomogram to predict the prognostic risk of pretreatment diffuse large B cell lymphoma patients. Eur Radiol 33:3354–3365. https://doi.org/10.1007/s00330-022-09301-5
139. Didion P, Crombé A, Dabadie A, et al (2022) Emergency whole-body CT scans in pediatric patients with trauma: patterns of injuries, yield of dual-phase scanning, and influence of second read on detection of injuries. Eur Radiol 32:8473–8484. https://doi.org/10.1007/s00330-022-08878-1
140. Ding W, Gu Y, Wu H, et al (2022) Mediastinal shift angle (MSA) measurement with MRI: a simple and effective tool for prenatal risk stratification in fetuses with congenital diaphragmatic hernia. Eur Radiol 33:1668–1676. https://doi.org/10.1007/s00330-022-09142-2

**Supplementary information 4:** Table of study characteristics.

| **Study** | **Clinical area assessed** | **Outcome assessed** | **Imaging modality** | **Type of model** | **Cited TRIPOD guidelines?** |
| --- | --- | --- | --- | --- | --- |
| **Ramtohul 2022** | Breast | Response post neoadjuvant therapy | MRI | Logistic | N |
| **Yang 2022** | Cardiac | Adverse cardiac events | CT | Cox | N |
| **Hahn 2022** | Thoracic | Fibrosis progression | MRI | Logistic | N |
| **Bhatt 2022** | Thoracic | Survival | CT | Cox | N |
| **Loving 2022** | Breast | Haematoma risk | Core needle biopsy | Logistic | N |
| **Lee 2022** | Breast | Second breast cancer risk | MRI | Cox | N |
| **Figliozzi 2022** | Cardiac | Sustained ventricular tachycardia, sudden cardiac death, or unexplained syncope | MRI | Cox | N |
| **Bian 2022** | HPB | Survival | CT | Cox | Y |
| **Tsakok 2022** | Thoracic | Hospital outcomes | CT | Linear | N |
| **Tran 2022** | Breast | Future breast cancer risk | Mammography | Cox | N |
| **Mauger 2022** | Cardiac | 10 year cardiovascular event rate | MRI | Linear | N |
| **Wang 2022** | Cardiac | Sudden cardiac death risk at 5 years | PET/MRI | Linear | N |
| **Lee 2022** | GI | 10 year adverse outcomes (death, cardiovascular events, fragility fractures) | CT | Logistic | N |
| **Kitao 2022** | HPB | Overall survival | CT, MRI | Unknown | N |
| **Fukutomi 2022** | Neurological | Clinical response to thrombectomy | MRI | Logistic | N |
| **Lee 2022** | Thoracic | Progression, lung cancer, mortality at 10 years | CT | Cox | N |
| **Kolossvary 2022** | Thoracic | Mortality at 30 days | Radiograph | Logistic | N |
| **Li 2022** | Cardiac | Sudden cardiac death, all cause mortality | MRI | Cox | N |
| **Shafaat 2022** | HPB | Mortality | CT | Cox | N |
| **Lee 2022** | Thoracic | Severe pneumonia on a CT score and clinical severity | CT | Logistic | N |
| **Kim 2022** | Thoracic | Overall survival | Radiograph | Cox | N |
| **Feng 2022** | HPB | Progression free survival, HCC subtype and immune profile | CT | Cox | N |
| **D’Amore 2022** | MSK | Fragility fracture risk | Radiograph | Logistic | N |
| **Diaz 2022** | Thoracic | Pulmonary exacerbation risk | CT | Logistic, linear | N |
| **Chen 2022** | Cardiac | Cardiac events | CT | Cox | N |
| **Jiang 2022** | HPB | Recurrence free survival | MRI | Logistic | Y |
| **Anzai 2023** | Head and Neck | Mortality | CT, MRI, PET/CT | Cox | N |
| **Lin 2023** | HPB | Local disease progression | CT | Cox | N |
| **Li 2023** | Cardiac | Sudden cardiac death | MRI | Cox | N |
| **Ghanbari 2023** | Cardiac | Adverse cardiac events | MRI | Cox | N |
| **Yeom 2023** | HPB | Pelvic/extra-hepatic metastases | CT | Cox | N |
| **Iravani 2023** | Miscellaneous | Overall survival | PET/CT | Cox | N |
| **Park 2023** | Thoracic | Overall survival, recurrence free survival | CT | Cox | N |
| **Zheng 2023** | Head and Neck | Disease progression | US | Cox, logistic | N |
| **Ragusi 2023** | Breast | Overall survival, recurrence free survival, disease free survival | MRI | Cox | N |
| **Park 2023** | Breast | Overall survival, recurrence free survival | MRI | Cox | N |
| **Xia 2023** | HPB | Overall survival, recurrence free survival | CT | Logistic | N |
| **Seifert 2023** | GU | Overall survival | PET/CT | Cox | N |
| **Napoli 2023** | MSK | Pain severity, disability scores | CT | Cox, linear | N |
| **Hou 2023** | Neurological | New ischaemic lesions | MRI | Logistic | N |
| **Salem 2023** | Neurological | Failure of embolisation | CT | Logistic | N |
| **Baraghoshi 2023** | Thoracic | Emphysema progression | CT | Linear | N |
| **Wyss 2022** | Neurological | Clinical scores | MRI | Linear | N |
| **Tschopp 2022** | MSK | Pain within 6 months and other adverse events | Radiograph | Linear | N |
| **Xu 2022** | Neurological | 3 month modified Rankin score (clinical outcome) | MRI | Logistic | N |
| **Xu 2022** | Neurological | Modified Rankin score (clinical outcome) | MRI | Logistic | N |
| **Park 2022** | Neurological | Overall survival | MRI | Cox | N |
| **Yu 2022** | Cardiac | Major adverse cardiac events | CT | Cox | N |
| **Wrobel 2022** | Thoracic | Lymph node and ablation zone change in size | CT | Linear | N |
| **Fong 2022** | Breast | Axillary lymph node metastasis | US | Logistic | N |
| **Xu 2022** | Breast | Axillary lymph node metastasis | MRI | Logistic | N |
| **Goh 2022** | Breast | Altered surgical plan | Contrast enhanced mammography | Logistic | N |
| **Rud 2022** | GU | Lymph node and bone metastases | MRI | Logistic | N |
| **Jiao 2022** | HPB | Overall survival | CT | Cox | N |
| **Janacova 2022** | MSK | Cartilage repair | CT | Linear | N |
| **Wang 2022** | Neurological | Post-surgical outcomes | PET/CT, MRI | Logistic | N |
| **Didion 2022** | Paediatrics | Missed injury in a second read | CT | Logistic | N |
| **Liu 2022** | Head and Neck | Cure rate | US | Logistic | N |
| **Peng 2022** | HPB | Liver failure | CT | Logistic | N |
| **Bian 2022** | GI | Overall survival | CT | Cox | N |
| **Meng 2022** | HPB | Liver failure | MRI | Logistic | N |
| **Jang 2022** | HPB | Motion artifact | MRI | Logistic | N |
| **Park 2022** | Thoracic | Recurrence free survival | CT | Cox | N |
| **He 2022** | Thoracic | Overall survival | CT | Cox | N |
| **Chen 2023** | GI | Microsatellite instability, survival | CT | Logistic | N |
| **Park 2023** | Thoracic | Recurrent haemoptysis | IR | Cox | N |
| **Xu 2023** | Neurological | Malignant middle cerebral artery infarction | IR | Logistic | N |
| **Rutten 2023** | Neurological | EEG slow wave activity | MRI | Logistic | N |
| **Liu 2023** | Neurological | Overall survival | MRI | Cox | N |
| **Chen 2023** | Cardiac | Left ventricular aneurysm | MRI | Logistic | N |
| **Pu 2023** | Cardiac | Atrial fibrillation onset | MRI | Cox | N |
| **Yan 2023** | GI | Overall survival | CT | Cox | N |
| **Li 2023** | Thoracic | Multidrug resistance | CT | Logistic | N |
| **Hwangbo 2023** | Breast | Response to chemotherapy | MRI | Logistic | N |
| **Ocal 2023** | HPB | Progression to HCC | MRI | Logistic | N |
| **Seo 2023** | HPB | HCC recurrence, overall survival | MRI | Cox | N |
| **Chen 2023** | HPB | Overall survival, progression free survival | CT | Cox | N |
| **Mohajer 2023** | MSK | Muscle degeneration, osteoarthritis progression, knee replacement | MRI | Cox | N |
| **Wu 2023** | HPB | Liver transplant, liver related death | MRI | Logistic | N |
| **Morotti 2023** | Neurological | Haematoma expansion, poor outcome | CT | Logistic | N |
| **Yan 2023** | Head and Neck | Recurrence free survival, local progression, lymph node metastases | US RFA | Cox | N |
| **Zhang 2023** | Thoracic | Progression free survival | CT | Cox | N |
| **Jiang 2023** | Cardiac | Cardioembolic stroke | CT | Logistic | N |
| **Chen 2023** | Thoracic | Overall survival | CT | Cox | N |
| **Song 2023** | Neurological | Atrial fibrillation | CT | Logistic | N |
| **Muller 2023** | HPB | Overall survival | CT | Cox | Y |
| **Aarts 2023** | GU | Local tumour free progression | RFA, MWA | Cox | N |
| **Zhao 2023** | Head and Neck | Lymph node metastases | CT | Logistic | N |
| **Tordjman 2023** | MSK | Overall survival | CT, MRI | Cox | N |
| **Hoffman 2023** | GI | Overall survival, progression free survival | CT | Cox | N |
| **Pamminger 2023** | Cardiac | Major adverse cardiac events | MRI | Cox | N |
| **Cha 2023** | HPB | Overall survival | CT, MRI | Cox | N |
| **Tan 2023** | HPB | Variceal bleeding | MRI | Logistic | N |
| **Xiao 2023** | HPB | Overall survival | MRI | Logistic | N |
| **Yin 2023** | MSK | Adverse events | MRI guided focussed US surgery | Logistic | N |
| **Li 2023** | MSK | Screw loosening | CT | Logistic | N |
| **Ding 2023** | Paediatrics | Postnatal outcomes | MRI | Logistic | N |
| **Lee 2023** | HPB | Recurrence | MRI | Cox | Y |
| **Zhang 2023** | Thoracic | Overall survival, progression free survival | PET/CT | Cox | N |
| **Hu 2023** | Miscellaneous | Cancer risk | Radiograph | Logistic | N |
| **Chen 2023** | HPB | Recurrence | CT | Logistic | N |
| **Xu 2023** | Neurological | Infarct core | MRI | Logistic | N |
| **Zhao 2023** | HPB | Early recurrence | Contrast enhanced US | Logistic | N |
| **Zhang 2023** | GI | Overall survival, progression free survival | MRI | Cox | N |
| **Liu 2023** | HPB | Overall survival, disease free survival | MWA | Cox | N |
| **Jiang 2023** | Cardiac | Adverse events | MRI | Cox | N |
| **Yu 2023** | Cardiac | Microvascular complications | CT | Logistic | N |
| **Tada 2023** | HPB | All cause death, hospitalisation | MRI | Cox | N |
| **Keijzer 2023** | Neurological | Neurological recovery | MRI | Logistic | N |
| **Jiang 2023** | Head and Neck | Disease free survival, response to chemotherapy | MRI | Cox | N |
| **Oh 2023** | Thoracic | PPFE development | CT | Logistic | N |
| **Lu 2023** | Neurological | Stenosis progression, stroke | MRI | Logistic | N |
| **Rozenblum 2023** | Neurological | Progression free survival | PET | Cox | N |
| **Abdelrady 2023** | Neurological | Complete recanalisation functional independence | MRI | Logistic | N |
| **Maruzzelli 2023** | HPB | Survival | TIPS | Cox | N |
| **Xing 2023** | Neurological | Outcomes after endovascular therapy | CT | Logistic | N |
| **Huang 2023** | MSK | Intraoperative blood loss, embolisation degree | Embolisation | Logistic | N |
| **Chu 2023** | HPB | Local tumour progression | Embolisation | Cox | N |
| **Park 2023** | HPB | Overall survival, recurrence free survival | CT | Cox | N |
| **Seo 2023** | GI | Disease free survival | CT | Cox | N |
| **Han 2023** | Miscellaneous | Completeness of resection | CT, US | Cox | N |
| **Fan 2023** | HPB | Overall survival | Embolisation | Cox | Y |
| **Dongxiang 2023** | Head and Neck | Overall survival | PET/CT | Cox | N |
| **Park 2023** | GU | Biochemical recurrence | MRI | Logistic | N |
| **Usami 2023** | Cardiac | Periprocedural ischaemia | MRI | Logistic | N |
| **Zu 2023** | Cardiac | Anastomosis occlusion | CT | Logistic | N |
| **Xi 2023** | Cardiac | Procedural success | CT | Logistic | N |
| **Yu 2023** | Cardiac | Major adverse cardiac events | CT | Cox | N |
| **Li 2023** | Thoracic | Tumour grade | CT | Logistic | Y |
| **Mascalchi 2023** | Thoracic | Overall mortality, cardiovascular mortality | CT | Cox | N |
| **Nam 2023** | Thoracic | Overall survival | CT | Cox | N |
| **Wang 2023** | GU | Overall survival, progression free survival | CT | Cox | N |
| **Li 2023** | Miscellaneous | Progression free survival | PET/CT | Cox | N |
| **Gou 2023** | HPB | Survival | TIPS | Cox | N |
| **Cai 2023** | HPB | Decompensation, transplant free survival | MRI | Cox | N |
| **Park 2023** | HPB | Recurrence free survival | MRI | Cox | N |
| **Wang 2023** | HPB | Recurrence free survival | MRI | Cox | N |
| **Wu 2023** | HPB | Overall survival, recurrence free survival | MRI | Cox | N |
| **Moore 2023** | GI | Survival | PET/CT | Cox | N |
| **Sun 2023** | Head and Neck | Overall survival | MRI, CT | Cox | N |

**Supplementary information 5:**

Table of TRIPOD domain adherence for individual studies included in the systematic review. Domains 10c, 10e, 12, 13c, 17, 19a relate to “validation” were not assessed as none of the 140 studies represented true external evaluation by authors uninvolved with development of the original model.


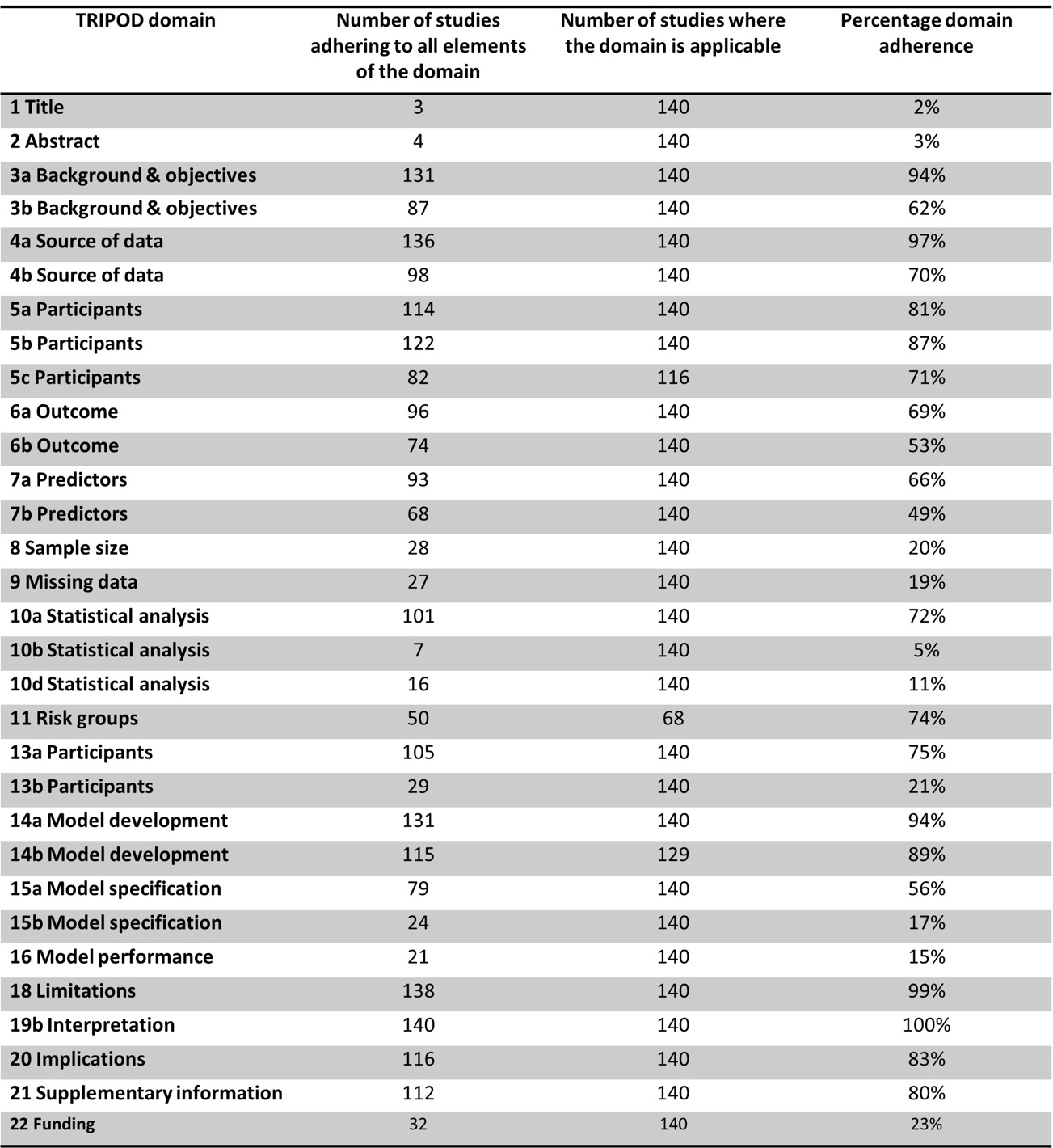

Supplement: tqaf174_Supplementary_Data [file tqaf174_supplementary_data.zip › Online Supplementary Material.docx]
